# Supplementary material for: Exclusive breastfeeding among beneficiaries of a nutrition enhancement programme and its associated factors in Ghana
Source: PLoS One. 2023 May 30;18(5):e0286546. doi: 10.1371/journal.pone.0286546 (PMC10228788; doi:10.1371/journal.pone.0286546)
Supplement: S1 File — (DOCX) [file pone.0286546.s001.docx]

**Table 1: Sample size distribution**

| **Facility** | **Sample size obtained** | **Percentage (%)** |
| --- | --- | --- |
| Dungu Health Centre (HC) | 40 | 11.8 |
| Kalpohin HC | 60 | 17.7 |
| Kanvilli HC | 54 | 15.6 |
| Nyohini HC | 61 | 18 |
| Reproductive and Child HC | 64 | 18.9 |
| Tampe Kukuo HC | 60 | 17.7 |

**Bivariate analysis of factors associated with exclusive breastfeeding**

The chi-square test revealed that sex of child (P=0.04) and child being sick within two weeks to the survey (P=0.028) were significantly associated with exclusive breastfeeding. Similarly, maternal ethnicity (P=0.002), occupation (P=0.02), religious affiliation (P=0.001), maternal education status (P<0.001) and household water source (P=0.008) were significantly associated with exclusive breastfeeding, (Table 2).

**Table 2: Bivariate analysis of factors associated with exclusive breastfeeding**

| **Variables** |  | **Exclusive breastfeeding** | | Test statistic |
| --- | --- | --- | --- | --- |
|  | **N** | **No** | **Yes** |  |
|  |  | Frequency  (%) | Frequency  (%) |  |
| **Maternal age categories(Years)** |  |  |  |  |
| 18-23 | 106 | 34(32.1) | 72(67.9) | P = 0.142 |
| 24-29 | 140 | 27(19.3) | 113(80.7) |  |
| 30-35 | 71 | 19(26.8) | 52 (73.2) |  |
| ≥ 36 | 10 | 3(30.0) | 7(70.7) |  |
| **Ethnic category** |  |  |  |  |
| Dagombas | 243 | 72(29.6) | 171(70.4) | X^2^ = 9.01**  P = 0.003 |
| Others (Akan, Chokosi, Ewe, Frafra, Fulani, Ga, Gonja, Hausa, Mamprusi and Waala) | 84 | 11(13.1) | 73(86.9) |  |
| **Maternal educational status** |  |  |  |  |
| None | 131 | 60(45.8) | 71(54.2) | X^2^ = 52.1  P < 0.001 |
| Moderate | 113 | 18(15.9) | 95(84.1) |  |
| High | 83 | 5(6.0) | 78(94.0) |  |
| **Marital status** |  |  |  |  |
| Married | 308 | 77(25.0) | 231(75.0) | P= 0.587 |
| Single | 19 | 6(31.6) | 13(68.4) |  |
| **Religious affiliation** |  |  |  |  |
| Christianity | 52 | 3(5.9) | 48(94.1) | X^2^= 11.91**  P < 0.001 |
| Islam | 287 | 80(29.0) | 196(71.0) |  |
| **Water source** |  |  |  |  |
| Well | 23 | 12(52.2) | 11(47.8) | X^2^ = 9.81**  P = 0.002 |
| Pipe-borne water | 304 | 71(23.4) | 233(77.1) |  |
| **Age of child (months)** |  |  |  |  |
| 0-4 | - | - | - | X^2^ = 0.07  P =0.789 |
| 5-12 | 185 | 48(25.9) | 137(74.1) |  |
| 13-24 | 142 | 35(24.6) | 107(75.4) |  |
| **Sex of child** |  |  |  |  |
| Female | 161 | 33(20.5) | 128(79.5) | X^2^= 3.997**  P = 0.046 |
| Male | 166 | 50(30.1) | 116(69.9) |  |
| **Currently breastfeeding** |  |  |  |  |
| No | 91 | 27(29.7) | 64(70.3) | X^2^ = 2.17  P = 0.14 |
| Yes | 236 | 56(23.7) | 180(76.3) |  |


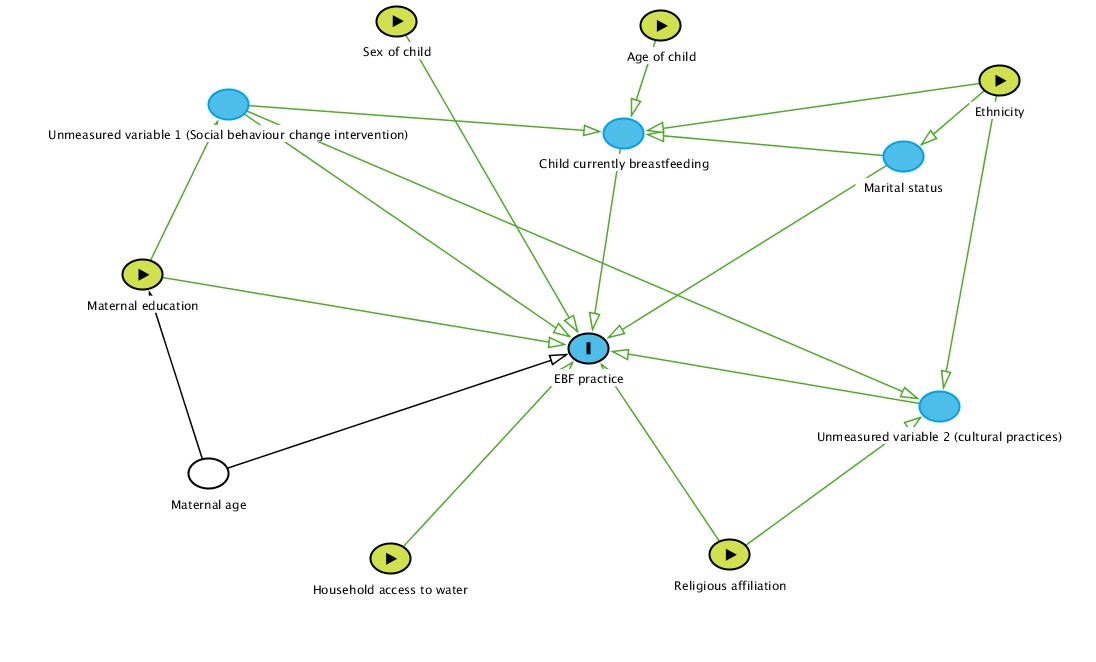


Figure 1: showing the exposures and how they relate to exclusive breastfeeding practice.

As shown in figure one, variables with small P-values were treated as the exposures (sex of child, age of child, ethnicity, religious affiliation, maternal education and household access to water) and model against EBF practice. Other unmeasured variables such as the SBCC and cultural practices were included to explain the pathways.
