# Supplementary material for: Exclusive breastfeeding among beneficiaries of a nutrition enhancement programme and its associated factors in Ghana
Source: PLoS One. 2023 May 30;18(5):e0286546. doi: 10.1371/journal.pone.0286546 (PMC10228788; doi:10.1371/journal.pone.0286546)
Supplement: Attachment — Submitted filename: Response to reviewers 05162023.docx [file pone.0286546.s003.docx]

 Response to Reviewers

We are grateful for the helpful comments and we have adequately addressed them.

| **Additional Editor Comments** | **Responses** |
| --- | --- |
| **Editor** |  |
| Thank you for your revisions! However, the conclusion still needs revision. Please note the conclusion is not just summary of results. You can include key observations with some future implications. | Response: We thank the Editor for the additional comments. We have revised the conclusion session of the abstract and main manuscript including possibly future implications. |
